# A comparative study of smart nanoformulations of diethyldithiocarbamate with Cu_4_O_3_ nanoparticles or zinc oxide nanoparticles for efficient eradication of metastatic breast cancer

**DOI:** 10.1038/s41598-023-30553-8

**Published:** 2023-03-02

**Authors:** Marwa M. Abu-Serie, Eisayeda Zeinab A. Abdelfattah

**Affiliations:** 1 Medical Biotechnology Department, Genetic Engineering and Biotechnology Research Institute, (GEBRI), City of Scientific Research and Technological Applications (SRTA-City), New Borg El‑Arab City, Alexandria, 21934 Egypt; 2 Animal House Unit, Medical Technology Center, Medical Research Institute, Alexandria University, Alexandria, Egypt

**Keywords:** Breast cancer, Cancer therapy, Pharmaceutics

## Abstract

Metastatic tumor is initiated by metastatic seeds (cancer stem cells “CSCs”) in a controlled redox microenvironment. Hence, an effective therapy that disrupts redox balance with eliminating CSCs is critical. Diethyldithiocarbamate (DE) is potent inhibitor of radical detoxifying enzyme (aldehyde dehydrogenase “ALDH”1A) causing effective eradication of CSCs. This DE effect was augmented and more selective by its nanoformulating with green synthesized copper oxide (Cu_4_O_3_) nanoparticles (NPs) and zinc oxide NPs, forming novel nanocomplexes of CD NPs and ZD NPs, respectively. These nanocomplexes exhibited the highest apoptotic, anti-migration, and ALDH1A inhibition potentials in M.D. Anderson-metastatic breast (MDA-MB) 231 cells. Importantly, these nanocomplexes revealed more selective oxidant activity than fluorouracil by elevating reactive oxygen species with depleting glutathione in only tumor tissues (mammary and liver) using mammary tumor liver metastasis animal model. Due to higher tumoral uptake and stronger oxidant activity of CD NPs than ZD NPs, CD NPs had more potential to induce apoptosis, suppress hypoxia-inducing factor gene, and eliminate CD44^+^CSCs with downregulating their stemness, chemoresistance, and metastatic genes and diminishing hepatic tumor marker (α-fetoprotein). These potentials interpreted the highest tumor size reduction with complete eradicating tumor metastasis to liver in CD NPs. Consequently, CD nanocomplex revealed the highest therapeutic potential representing a safe and promising nanomedicine against the metastatic stage of breast cancer.

## Introduction

Breast cancer is the second leading cause of cancer death in women globally. Breast cancer mortality is attributed to metastatic growth, not the primary tumor^1,2^. Liver is the most common and first metastasis target organ of breast tumor^3^. The breast cancer liver metastasis is triggered by multiple factors in breast cancer stem cell (seed) and liver microenvironment (soil) under a tightly controlled redox balance via lowering cellular reactive oxygen species (ROS) and elevating antioxidant mediators (e.g., glutathione)^4,5^. One of the key factors in breast cancer cell metastasis (invasion and adhesion) is CD44, which is expressed on cancer stem cells (CSCs)^5,6^. These CSCs have a high expression of aldehyde dehydrogenase (ALDH) 1-maintained non-ntoxic reactive radical levels to mediate cell invasion, and apoptosis resistance^5,7^. The elevated ALDH1 level in breast cancer patients is associated with chemoresistance and metastasis. Hence, ALDH1 activates stemness pathways (Notch and Wnt/β-catenin) and hypoxia-inducible factor (HIF)1α/vascular endothelial growth factor (VEGF) pathway^8,9^. The main factor which modulates the microenvironment of metastatic CSCs is HIF1α by reducing the mitochondrial oxidative stress and enhancing the antioxidant synthesis, thereby promoting chemoresistance^10,11^. Also, HIF and its related genes (e.g., vascular endothelial growth factor, VEGF and TWIST) maintain angiogenesis and the activated expression of matrix metalloproteases (MMP), E-cadherin, and β-catenin^5,12^. The latter gene enhances the expression of anti-apoptotic, chemoresistance, MMP genes, and CD44 as well as activates Notch signaling-mediated upregulating stemness genes, which also contribute to chemoresistance^6,13,14^.

The short median survival rate among patients with metastatic breast cancer is due to the insensitivity to current endocrine therapy, chemotherapy, and immunotherapy^3^. Therefore, more research is needed to establish an effective treatment for metastatic breast cancer. Yao et al. found a correlation between high ALDH1 expression and the progression of invasive ductal carcinoma in breast cancer patients^15^. Furthermore, Croker et al. evidenced that knocking out ALDH1A1 inhibited the chemoresistance and metastasis of CD44^+^ breast cancer cells^9^. A previous study proved that the anti-metastatic potency of diethyldithiocarbamate (the main metabolite of FDA-approved ALDH1 inhibitor “disulfiram”) in cholangiocarcinoma cell lines via suppressing migration and adhesion^16^. Moreover, the proapoptotic efficacy of disulfiram (parent of diethyldithiocarbamate), as aldehyde dehydrogenase inhibitor, in collapsing CSCs with suppressing their epithelial-mesenchymal transition (EMT, a crucial step in mediating metastasis), was studied^17^. Interestingly, the chelating copper-diethyldithiocarbamate complex exhibited a stronger anticancer activity than its parent (disulfiram)^18,19^. Cvek et al. investigated the anti-breast cancer activity of three types of metal-diethyldithiocarbamate (DE) complexes, including copper(II)-DE, zinc(II)-DE, and nickel-DE. Copper-DE and zinc-DE chelating complexes, but not nickel-DE complex, were found to have powerful proteasome inhibition-dependent anticancer efficacy against in M.D. Anderson-metastatic breast (MDA-MB) 231 cells^20^. Nevertheless, the preclinical application of DE complex was limited due to its low solubility and less selectivity against cancer cells, which leads to toxicity towards normal cells^18^. Thus, nanoformulation of metal-DE complex is needed. Ren et al. prepared a nanocomplex of copper-DE, by loading it into organic phase-change nanomaterial comprising of stearic acid and lauric acid as the core and phospholipid as the shell. Then a photochemical agent was added to this nanocomplex to achieve a selective tumor targeting upon photothermal heating against breast cancer. This nanocomplex, which was accumulated selectively in breast tumor with little toxicity in normal tissues, exhibited high anticancer effect and anti-metastatic activity^18^. Several recent studies illustrated the potent proapoptotic activity of green synthesized copper oxide nanoparticles (NPs) and zinc oxide NPs against breast cancer cell lines^21–25^. The anticancer activity of such metal oxide NPs is primarily due to their pro-oxidant effects, which induce severe oxidative cellular damage^26^. Recently, a novel simple nanoformulation of DE containing green chemosynthesized-Cu_2_O NPs revealed higher oxidant effect-mediated anticancer activity with anti-migration potency than its traditional complex against lung, colon, liver and prostate cancer cells^27^.

In this current study, for the first time, DE was nanoformulated with green chemically synthesized Cu_4_O_3_ NPs or zinc oxide NPs for effective elimination of metastatic breast cancer cells. Hence, the apoptotic and anti-migration activity of these unique nanocomplexes of DE were investigated against the highly metastatic breast cancer cells (MDA-MB 231). Moreover, DE-nanocomplexes were evaluated for their pro-oxidant-mediated apoptotic activity and inhibitory efficacy on key mediators of anti-apoptosis, hypoxia, stemness, chemoresistance, angiogenesis, and metastasis in an orthotopic metastatic breast tumor animal model.

## Results

### Characterization of the prepared NPs

In this study, DE was nanoformulated with green chemically synthesized CO NPs or ZO NPs. These prepared metal oxide NPs have sizes about 112.5 ± 2.5 nm and 101 ± 4.0 nm, respectively (Supplementary Fig. 1), with zeta potentials about 16.5 ± 1.5 mV and − 12.25 ± 0.75 mV, respectively (Supplementary Fig. 2). The nanoformulations of the produced CD NPs and ZD NPs were affirmed by zetasizer-assessed sizes of 156.5 ± 3.5 nm and 206.5 ± 2.5 nm, respectively (Supplementary Fig. 1), with zeta potentials of − 4.65 ± 0.45 mV and − 14.65 ± 0.65 mV, respectively (Supplementary Fig. 2).

**Figure 1:**
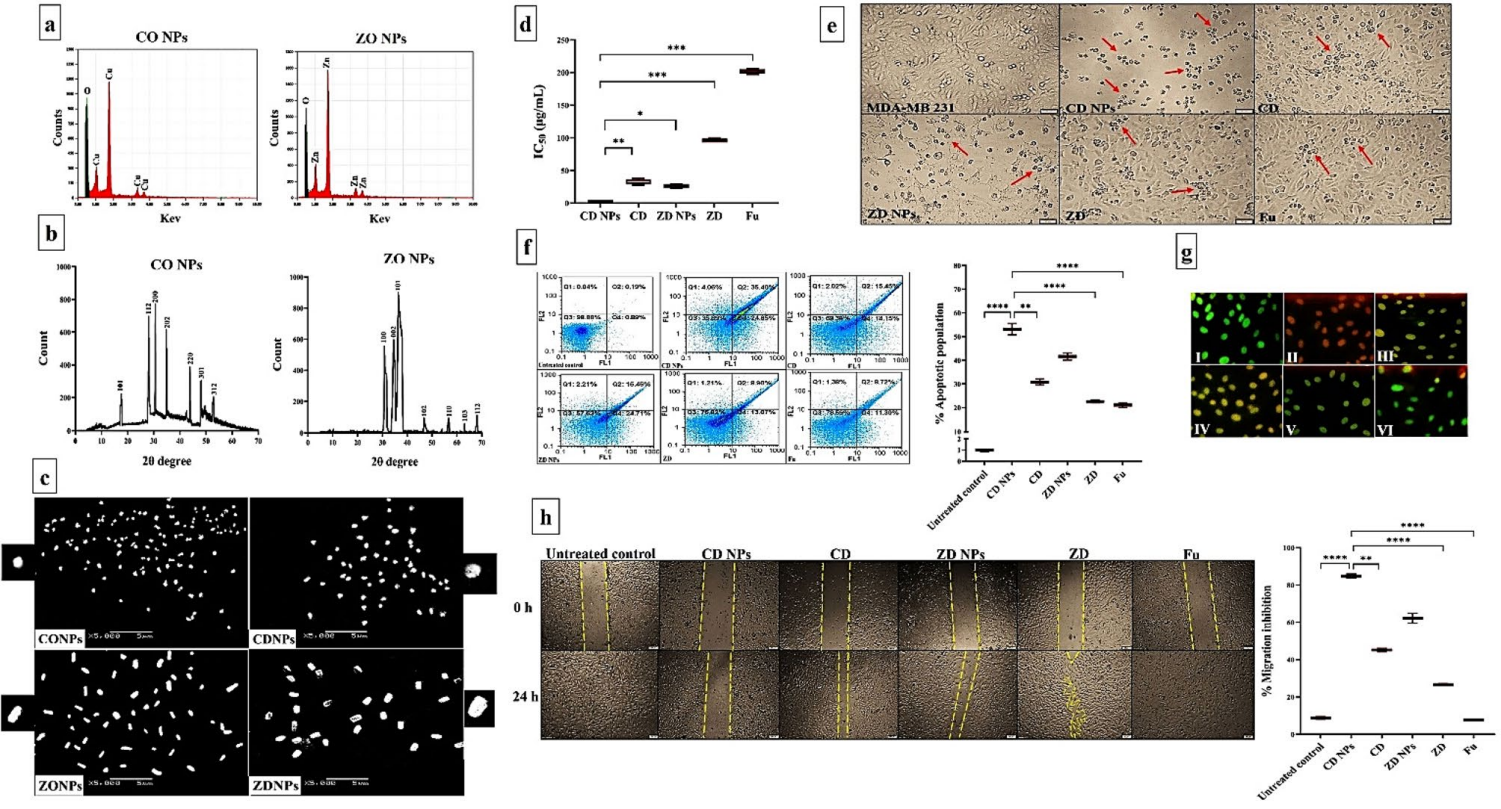
Characterization of the prepared NPs and anticancer impact on MDA-MB 231 cells. (**a**) Elemental charts of Cu and O in copper oxide (CO) NPs as well as Zn and O in zinc oxide (ZO) NPs using energy dispersive X-ray analysis (EDX). (**b**) X-ray diffractometer (XRD) patterns for CO NPs and ZO NPs. (**c**) scanning electron microscope images of CO NPs and ZO NPs as well as their corresponding nanocomplexes with diethyldithiocarbamate (CD NPs and ZD NPs, respectively) at magnification, × 5000; scale bar, 5 µm. For more declaration, individual nanoparticle of each prepared type was showed at maximum magnification. (**d**) MTT results in the term of IC_50_ values of CD NPs, CD, ZD NPs, ZD, and 5-fluorouracil (Fu) with (**e**) morphological alterations of these treated MDA-MB 231 cells (Red arrows refer to apoptotic cells) compared to the untreated control cells. (Magnification, × 100; scale bar, 50 µm). (**f**) Apoptotic activity as illustrated by flow cytometry dot plots of the untreated MDA-MB 231 and CD NPs-, CD-, ZD NPs-, ZD-, and Fu-treated cells with the total percentage of apoptosis after staining with annexin V/propidium iodide as well as (**g**) fluorescence microscopic images for the untreated and treated MDA-MB 231 after incubation with acridine orange and ethidium bromide (Green, yellow and orange-red fluorescence reflect healthy, early apoptotic, and late apoptotic cell population, respectively) (Magnification, × 100; scale bar, 50 µm). (**h**) Anti-migration activity as showed by microscopic pictures of the wound area (yellow dashed line-labeled) in the untreated and treated MDA-MB 231 cells at 0 and 24 h (Magnification, × 40; scale bar, 200 µm) with the percentage of migration inhibition. Data are demonstrated as mean ± SEM. CD NPs were compared to other tested compounds, considering significantly different at p < 0.05*, < 0.01**, and < 0.001***.

Furthermore, the prepared CO NPs and ZO NPs were characterized using EDX that illustrated the presence of intense signals of Cu (57.69%) and Zn (66.67%), respectively, with oxygen intensities of 42.31% and 33.33%, respectively (Fig. 1a). XRD patterns of CO NPs and ZO NPs correspond to tetragonal Cu_4_O_3_ (JCPDS file no. 49-1830) and ZnO (JCPDS file no. 36-1451), respectively (Fig. 1b). Micrographs of SEM (Fig. 1c) show a quasi-spherical structure for CO NPs and its nanocomplex of CD NPs, while ZO NPs and ZD NPs have a rod shape.

### In vitro anticancer activity

#### Cytotoxic, proapoptotic, and anti-migratory impact against MDA-MB 231 cells

Cytotoxicity of the prepared NPs (CO NPs, ZO NPs, CD NPs, and ZD NPs) was determined, in the term of IC_50_, compared to CD, ZD, DE, copper chloride, zinc chloride, and standard chemotherapy (fluorouracil “Fu”) against MDA-MB 231 cells (Fig. 1d). Based on the lowest IC_50_ indicating the highest anticancer activity (Fig. 1d), nanocomplexes of CD NPs and ZD NPs exhibited the strongest growth inhibitory effect against MDA-MB 231 cells compared to their corresponding traditional complexes (160.78 ± 3.55 μg/mL and 173.99 ± 5.14 μg/mL, respectively), Fu (184.19 ± 3.93 μg/mL), and DE (201.48 ± 1.87 μg/mL). The IC_50_ values of copper chloride and zinc chloride could not be calculated due to their maximum utilized concentration did not reach 50% death in the treated MDA-MB 231. Importantly, IC_50_ of CD NPs (31.93 ± 5.14 μg/mL) was significantly lower than that of ZD NPs (95.98 ± 1.69 μg/mL). Figure 1e illustrates the highest drastic alterations in the morphology of CD NPs-treated MDA-MB 231 cells compared to CD-, ZD NPs-, ZD-, and Fu-treated cells.

Flow cytometry analysis (Fig. 1f) of the apoptotic population (%) for the treated MDA-MB 231 cells clarified that both nanocomplexes (> 40%) were the highest compared to CD (30.81%), ZD (22.58%), and Fu (21.04%). As shown in Fig. 1f, the proapoptotic effect of CD NPs (55.30 ± 4.75%) was significantly higher than that of ZD NPs (41.60 ± 1.44%). These results were confirmed by fluorescence microscope after staining with EB/AO, where green, yellow and reddish-orange fluorescence nuclei reflect healthy, early apoptotic, and late apoptotic cells, respectively (Fig. 1g). Subsequently, the late apoptotic population was only observed in CD NPs-treated MDA-MB 231 cells, whereas the other treated cells refer to the early apoptosis stage (Fig. 1f,g).

Regarding wound healing assay (Fig. 1h), nanocomplexes and traditional complexes revealed a 40-fold higher anti-migratory effect than Fu. Moreover, both nanocomplexes demonstrated higher anti-migration potency than their corresponding conventional complexes in the treated MDA-MB 231 cells. CD NPs inhibited cell migration by 84.83 ± 1.09%, whereas ZD NPs suppressed migration by 62.27 ± 2.59% (Fig. 1h).

#### Inhibitory potential on ALDH1 activity

**Figure 2:**
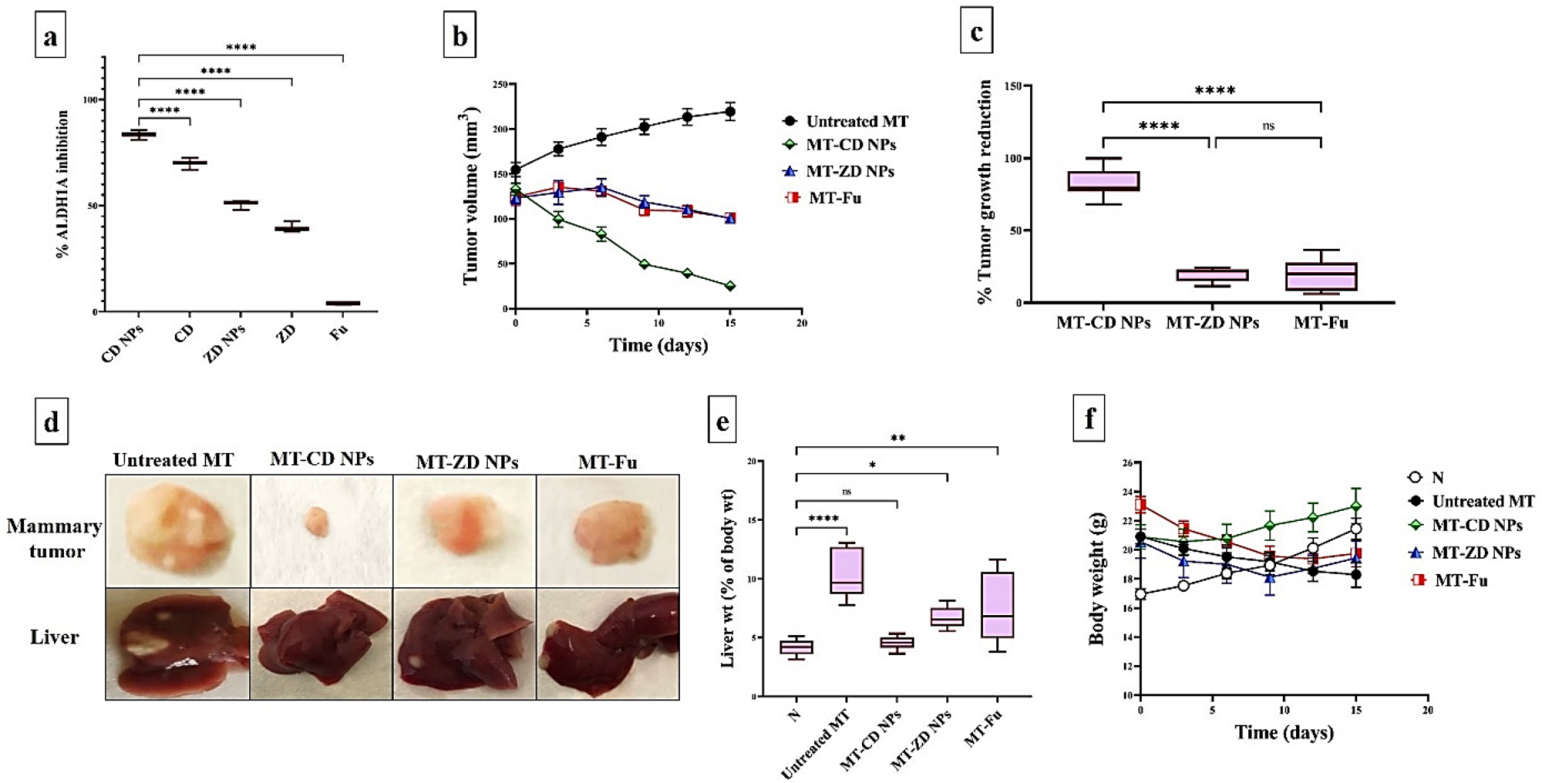
In vitro ALDH1A inhibitory potential and inhibitory impact on tumor growth using orthotopic mammary tumor animal model. (**a**) The relative ALDH1A inhibition in the treated MDA-MB 231 cells. (**b**) The recorded mammary tumor volume using caliper with (**c**) the reduction in mammary tumor weight that was measured, at the end of experiment, in all three treated groups compared to the untreated mammary tumor-bearing mouse group (MT). Data are demonstrated as mean ± SEM. MT-CD NPs group was compared to other groups, considering significantly different at p < 0.05*, < 0.01**, and < 0.001***. (**d**) Macroscopic images of mammary tumors and liver tumor nodules of the untreated and treated MT groups. (**e**) Liver weight, relative to body weight, for untreated and treated MT groups compared to the healthy control group (N). (**f**) Body weight for untreated and treated MT groups compared to N group. All values are demonstrated as mean ± SEM. Healthy (N) untreated group was compared to other groups, considering significantly different at p < 0.05*, < 0.01**, and < 0.001***.

Figure 2a demonstrates that CD NPs had the strongest inhibitory ALDH1A potential (83.40%) compared to other prepared DE complexes and Fu. Notably, CDNPs were more effective than ZD NPs (69.86%) for suppressing ALDH1A activity. On the other hand, Fu exhibited the lowest inhibition potency (< 4%) for ALDH1A.

### Therapeutic potency against metastatic mammary tumor (in vivo)

#### Inhibitory impact on tumor volume and weight

The anti-tumor efficacy of the most active compounds (CD NPs and ZD NPs), in comparison with Fu, was studied in vivo using an orthotopic mammary tumor (MT) model. During 2 weeks of treatment, six injections of CD NPs were able to decrease the tumor size mean, from 118.74 to 25.11 mm^3^, significantly more than ZD NPs and Fu. In contrast to CD NPs-treated MT group (MT-CD NPs), ZD NPs-treated MT group (MT-CD NPs), and Fu-treated MT group (MT-Fu NPs), the tumor size of the untreated MT group increased from 113.20 to 219.38 mm^3^ during 2 weeks (Fig. 2b). The tumor weighing revealed that MT-CD NPs group had the highest reduction (81.11 ± 4.07%) in tumor growth, compared to 18.42 ± 2.44% and 18.99 ± 4.11% for MT-ZD NPs and MT-Fu groups, respectively (Fig. 2c). Additionally, macroscopic images of mammary tumors (Fig. 2d) support the superior inhibitory potential of CD NPs on mammary tumor growth and metastasis, as evidenced by the disappearance of liver tumor nodules, compared to ZD NPs and Fu as well as the untreated MT. Furthermore, the untreated MT group had the largest weights of liver organ relative to its corresponding body weight (7.724 ± 1.69%) compared to all treated MT groups and N group (Fig. 2e). This figure also shows no significant difference between liver index of N group and MT-CD NPs group was recorded, but there is statistical variation with MT-ZD NPs and MT-Fu groups. According to mice body weights, the highest reduction in body weight was observed in the untreated MT group compared to all treated groups and N group. Particularly, body weight of MT-CD NPs group was stable as compared to healthy N group (Fig. 2f).

#### Histological analysis of the therapeutic efficacy of nanocomplexes

**Figure 3:**
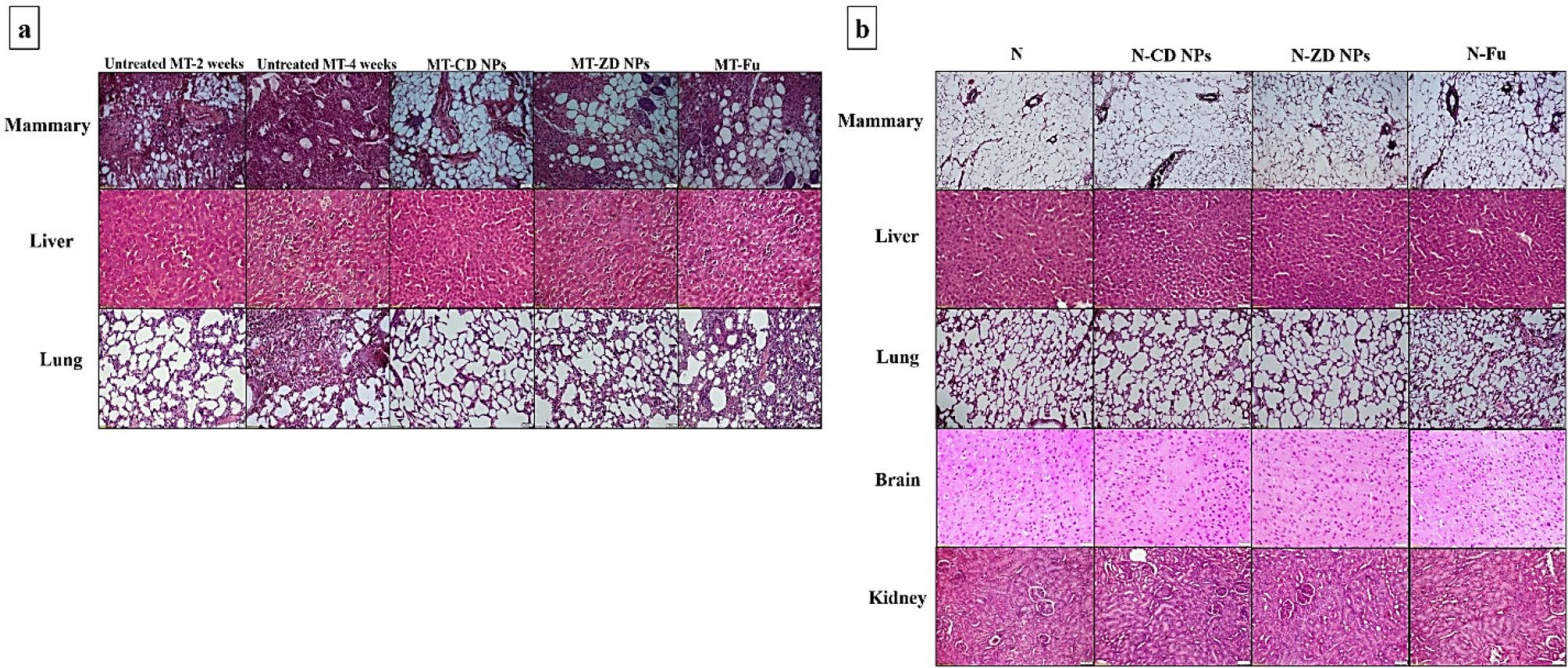
Histological analysis of anti-tumor potential and toxicity on normal tissues. (**a**) Hematoxylin and eosin (H&E)-stained tissue sections of the untreated mammary tumor-bearing mouse group (MT) after 2 weeks and 4 weeks of Ehrlich cell orthotopic injection (showing the mixed ductal and lobular mammary carcinoma with invasion to liver and lung hyperplasia) as well as the treated MT groups. (**b**) H&E-stained tissue sections of healthy untreated group (N) and treated N groups (Magnification, × 200; scale bar, 50 µm).

After 2 weeks of orthotopic injection of Ehrlich cells, the incidence of a metastatic mammary tumor was confirmed by H&E staining mammary, liver, and lung tissues that illustrated the mixed ductal and lobular mammary carcinoma, hepatic tumor cells, and normal lung, respectively (Fig. 3a). Following 2 weeks of treatment with nanocomplexes or Fu, H&E staining declared that CD NPs had the best therapeutic potential of CD NPs on mammary tumor with complete suppression of metastasis to liver, compared to ZD NPs and Fu. Unlike the untreated MT and MT-Fu, both nanocomplexes completely halted lung hyperplasia (Fig. 3a). Moreover, H&E stained mammary, liver, lung, brain, and kidney tissues of N-CD NPs, N-ZD NPs, and N-Fu groups revealed no histological alterations when compared to N group (Fig. 3b).

#### Selective accumulation of nanocomplexes in tumor tissues

**Figure 4:**
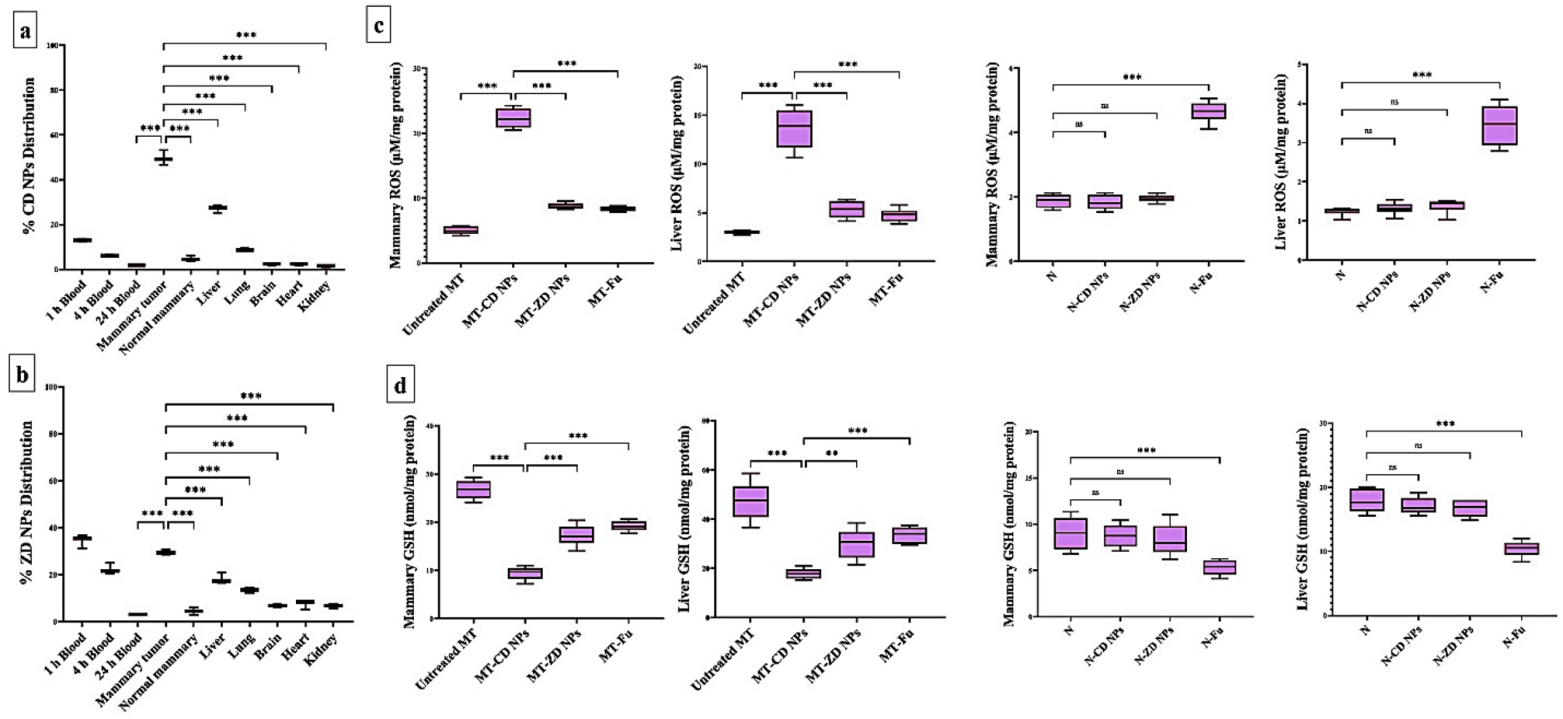
Nanocomplexes’ selective biodistribution in tumor tissues and selective ROS elevation with GSH depletion impacts. Selective biodistribution of (**a**) CD NPs and (**b**) ZD NPs in blood and tissues. All values are demonstrated as mean ± SEM. Mammary tumor was compared to other tissues, considering significantly different at p < 0.05*, < 0.01**, and < 0.001***. (**c**) ROS level (μM/mg protein) and (**d**) GSH content (nmol/mg protein) in mammary tumor and liver of the untreated mammary tumor-bearing mouse group (MT), CD NPs-treated MT group (MT-CD NPs), MT-ZD NPs group, and MT-Fu group compared to normal mammary and liver tissues of healthy control (N) untreated group, CD NPs-treated N group (N-CD NPs), N-ZD NPs, and N-Fu. All values are demonstrated as mean ± SEM. MT-CD NPs group was compared to other groups, considering significantly different at p < 0.05*, < 0.01**, and < 0.001***.

The biodistribution of nanocomplexes was studied, separately, by i.v. injection of CD NPs and ZD NPs into MT mice, after 4 weeks of Ehrlich cell suspension injection. The results of atomic absorption spectroscopy demonstrated that 13.00% and 6.187% of CD NPs and 34.48% and 22.34% of ZD NPs remained in blood at 1 h and 4 h post injections, respectively, in the treated MT mice (Fig. 4a,b). After 24 h, CD NPs and ZD NPs distributed mostly (49.71% and 32.25%, respectively) in mammary tumor, followed by liver (27.10% and 18.22%, respectively) and lung (8.930% and 13.48%, respectively). The other investigated tissues (brain, heart and kidney) and blood contained the lowest amounts of CD NPs (< 2.5%) and ZD NPs (< 7%) as shown in Fig. 4a,b.

#### Selective pro-oxidant effect of nanocomplexes

In mammary tissues of the treated MT groups, CD NPs, ZD NPs, and Fu enhanced ROS generation by 4.431, 1.736, and 1.656 folds, respectively, relative to the untreated MT. Also, MT-CD NPs, MT-ZD NPs, and MT-Fu groups showed an increase in hepatic ROS levels by 2.708-, 1.060-, and 1.000-folds, respectively, compared to the untreated MT (Fig. 4c). This ROS elevation was associated with GSH depletion, CD NPs (~ threefold) outperforming ZD NPs (~ 1.5-fold) and Fu (~ 1.4-fold) in diminishing GSH level in both mammary tumor and liver tissues relative to the untreated MT (Fig. 4d). In contrast to Fu, both nanocomplexes did not cause any significant elevation of ROS or reduction in GSH levels in the normal mammary and liver tissues of N-CD NPs and N-ZD NPs groups relative to N group (Fig. 4c,d).

#### Immunohistochemical investigation of the therapeutic efficacy of nanocomplexes

**Figure 5:**
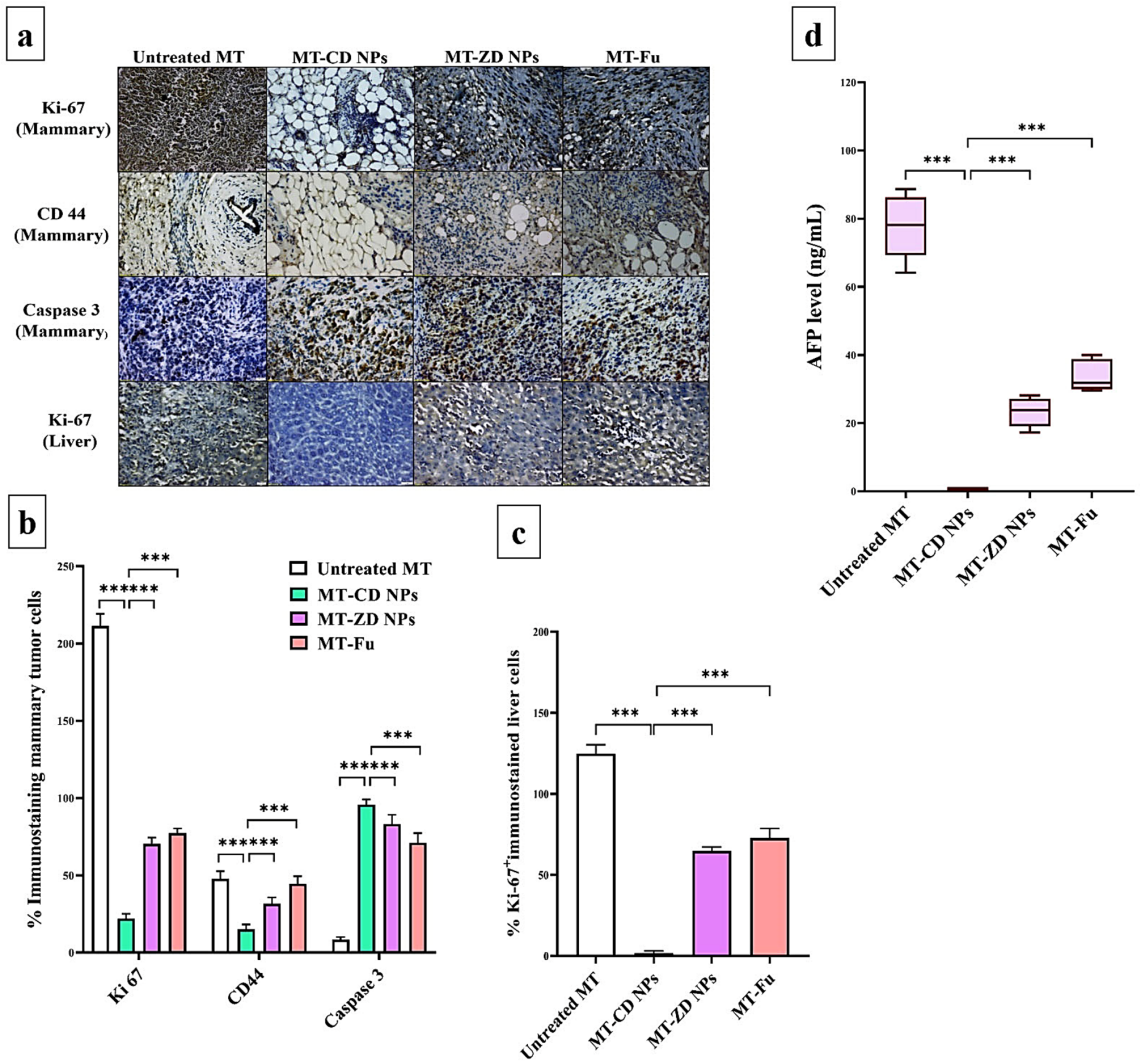
Immunohistochemical analysis and biochemical detection of anti-metastatic tumor potential. (**a**) Microscopic images of Ki-67, CD44, and caspase 3-immunostained mammary tumor as well as Ki-67-immunostained liver sections of the untreated mammary tumor-bearing mouse group (MT), CD NPs-treated MT group (MT-CD NPs), MT-ZD NPs group, and MT-Fu group with (**b,c**) their percentages of positive immunostained tumor cells. (**d**) The blood level (ng/mL) of α-fetoprotein (AFP). All values are demonstrated as mean ± SEM. MT-CD NPs group was compared to other groups, considering significantly different at p < 0.05*, < 0.01**, and < 0.001***.

The immunostaining tumor tissues of mammary and liver with Ki-67 (Fig. 5a–c) illustrated that CD NPs exhibited the highest suppressive potential on this proliferative marker, by 9.61 and 69.45-folds, respectively, compared to ZD NPs (3.00 and 1.92-folds, respectively) and Fu (2.73 and 1.71-folds, respectively). Also, CD NPs demonstrated the highest anti-CSC and proapoptotic activities in mammary tumor, as evidenced by the minimum percentage of CD44^+^-stained cells (20.26 ± 2.19%) and the maximum percentage of caspase 3^+^-stained cells (95.71 ± 1.49%) compared to ZD NPs and Fu. There is no significant difference was recorded between Zn NPs and Fu in the terms of % Ki-67^+^-and caspase 3^+^-immunostained tumors. However, the percentage of CD44^+^-stained mammary tumor cells of MT-Zn NPs group was significantly lower than that of MT-Fu group, p < 0.05 (Fig. 5a–c).

#### Suppressive effect of nanocomplexes on liver tumor marker

The main liver tumor marker (α-fetoprotein) which was used to evaluate the anti-metastatic potential of the prepared nanocomplexes, was elevated abnormally in the untreated MT group by > 77 folds relative to the healthy N group. As shown in Fig. 5d, CD NPs can normalize α fetoprotein (AFP < 0.6 ng/mL), whereas its level was still high in MT-ZD NPs group (23.25 ± 1.94 ng/mL) and MT-Fu group (33.89 ± 2.08 ng/mL).

#### Regulatory effect of nanocomplexes on the expression of the main proapoptotic gene and oncogenes

**Figure 6:**
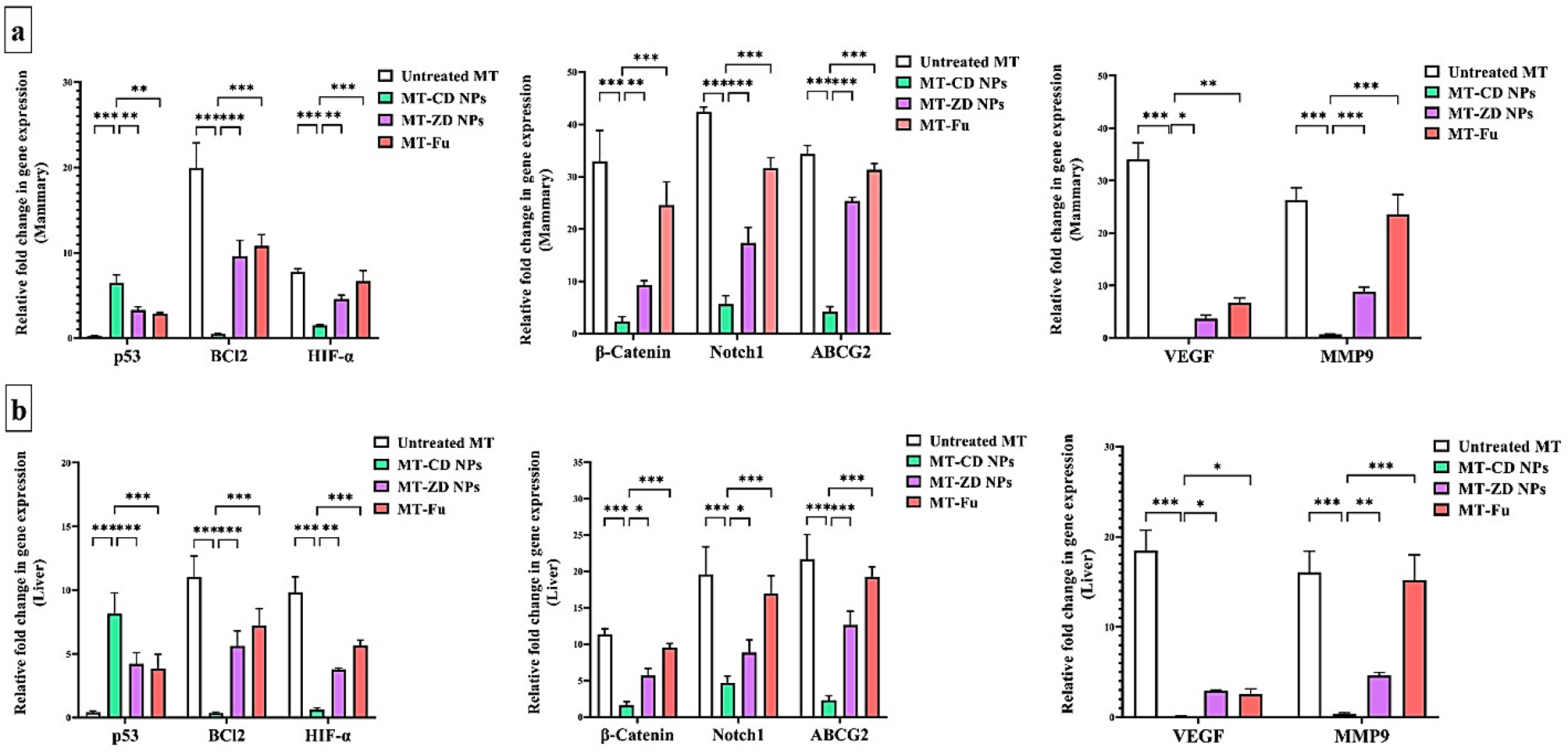
Regulatory impact on the expression of key apoptotic gene and oncogenes using qPCR. The relative gene expression of p53, BCl2, hypoxia-inducing factor (HIF)-α, β-catenin, Notch1, ATP Binding Cassette Subfamily G Member 2 (ABCG2), vascular endothelial growth factor (VEGF), and matrix metalloprotease (MMP)9 were quantified in (**a**) mammary tumor and (**b**) liver tissues of the untreated mammary tumor-bearing mouse group (MT), CD NPs-treated MT group (MT-CD NPs), MT-ZD NPs group, and MT-Fu group. All values are demonstrated as mean ± SEM. MT-CD NPs group was compared to other groups, considering significantly different at p < 0.05*, < 0.01**, and < 0.001***.

Figure 6a,b shows that CD NPs exhibited the highest activity for upregulating the expression of the main proapoptotic gene (p53), by 6.461 and 8.141-folds compared to ZD NPs (3.272 and 4.213-folds) and Fu (2.858 and 3.857-folds) in mammary tumor and liver tissues, respectively. There is no significant variation in the ability of ZD NPs and Fu to induce p53 expression. Furthermore, CD NPs had the highest potential to repress key anti-apoptotic gene (BCl2), HIF-α-driven hypoxic microenvironment, β-catenin- and Notch1-mediated stemness, ABCG2-dependent chemoresistance, VEGF-dependent angiogenesis, and MMP9-stimulated metastasis, in both mammary and liver tumor tissues. ZD NPs suppressed the above-mentioned oncogenes more effectively than Fu, excluding HIF, VEGF, and BCl2 expression which showed a comparable inhibitory effect between ZD NPs and Fu (Fig. 6a,b).

#### Safety of these nanocomplexes on liver, kidney and hematological parameters

For investigating the in vivo toxicity profile of these nanocomplexes, functional parameters of liver (ALT, AST, and albumin) and kidney (urea and creatinine) as well as CBC were assessed in N and MT groups that were treated with CD NPs and ZD NPs versus the untreated groups (Tables 1, 2). Table 1 reveals that both nanocomplexes did not cause any significant variation in the functional parameters of liver (ALT, AST, and albumin) or kidney (urea and creatinine) in either the treated N or treated MT groups, relative to the healthy N group. Meanwhile, Fu caused a substantial increase in liver function parameters, including ALT and AST, in N-Fu and MT-Fu groups. On the other hand, the untreated MT group demonstrated significant abnormal alterations in liver parameters, including elevation of ALT and AST with diminishing albumin levels, and a moderate increase in kidney functions, compared to healthy N group (Table 1).

**Table 1:** Liver and kidney functional parameters.

	N	N-CD NPs	N-ZD NPs	N-Fu	MT	MT-CD NPs	MT-ZD NPs	MT-Fu
ALT (U/L)	99.21 ± 3.15	96.99 ± 2.74	92.02 ± 0.63	108.4 ± 6.65	138.4 ± 4.68***	93.69 ± 3.07	110.1 ± 4.16	119.0 ± 2.03**
AST (U/L)	186.1 ± 5.87	177.0 ± 9.95	184.2 ± 7.61	208.3 ± 13.8	203.7 ± 4.33**	184.9 ± 7.48	199.2 ± 3.49	205.8 ± 3.47**
Albumin (g/dL)	3.989 ± 0.31	4.305 ± 0.26	4.166 ± 0.29	4.267 ± 0.26	3.040 ± 0.12**	4.323 ± 0.20	3.483 ± 0.06	3.360 ± 0.11*
Urea (mg/dL)	47.64 ± 3.04	48.25 ± 1.45	49.13 ± 1.01	49.38 ± 0.83	56.33 ± 4.65	44.65 ± 5.21	46.54 ± 2.68	49.62 ± 2.77
Creatinine (mg/dL)	1.844 ± 0.08	1.707 ± 0.19	1.644 ± 0.15	1.679 ± 0.14	1.969 ± 0.05	1.608 ± 0.12	1.757 ± 0.15	1.678 ± 0.16

*N* the untreated healthy control normal mouse group, *N-CD NPs* nanocomplex of Cu_4_O_3_ with diethyldithiocarbamate (DE)-treated normal mouse group, *N-ZD NPs* nanocomplex of ZnO with DE-treated normal mouse group, *N-Fu* 5-fluorouracil-treated normal mouse group, *MT* the untreated mammary tumor-bearing mouse group, *MT-CD NPs* CD NPs-treated MT group, *MT-ZD NPs* ZD NPs-treated MT, and *MT-Fu* Fu-treated MT group.

All values are demonstrated as mean ± SEM. N group was compared to other groups and considered significantly different at p < 0.05*, < 0.01**, and < 0.0001***.

**Table 2:** Hematological parameters.

	N	N-CD NPs	N-ZD NPs	N-Fu	MT	MT-DC NPs	MT-DZ NPs	MT-Fu
RBC								
RBC (10^6^/µL)	7.99 ± 0.09	8.13 ± 0.13	7.53 ± 0.47	7.10 ± 0.10	6.84 ± 0.16	8.04 ± 0.33	7.35 ± 0.34	6.33 ± 0.43
Hg (g/dL)	13.7 ± 0.70	14.2 ± 0.11	12.6 ± 0.40	13.3 ± 0.22	9.85 ± 0.75*	11.1 ± 0.35	10.8 ± 0.90	9.40 ± 1.20*
HCT (%)	35.4 ± 1.09	35.7 ± 0.29	33.1 ± 1.85	33.5 ± 0.48	26.2 ± 1.60*	29.2 ± 1.45	27.9 ± 2.20	25.3 ± 2.65*
MCV (fL)	34.6 ± 0.65	34.8 ± 0.37	34.6 ± 0.37	36.7 ± 0.45	38.2 ± 1.35	36.3 ± 0.25	37.8 ± 1.25	40.0 ± 1.45*
MCH (pg)	17.4 ± 0.40	17.4 ± 0.12	17.7 ± 0.40	18.4 ± 0.30	14.3 ± 0.75	16.8 ± 0.15	14.6 ± 0.55	14.8 ± 0.90
MCHC (g/dL)	39.1 ± 0.15	39.8 ± 0.15	39.4 ± 0.28	39.0 ± 0.09	37.5 ± 0.50	38.0 ± 0.65	38.6 ± 0.20	37.0 ± 1.00
RDW-CV (%)	13.2 ± 0.2	13.8 ± 0.15	15.3 ± 0.2	15.0 ± 0.25	21.9 ± 0.80*	16.4 ± 1.2	21.8 ± 3.15	20.2 ± 0.30*
RDW-SD fL)	21.4 ± 0.40	21.2 ± 0.25	24.2 ± 0.15	25.6 ± 0.51	30.2 ± 0.05*	28.6 ± 2.05	29.7 ± 3.40	29.1 ± 1.45*
WBC								
WBC (10^3^/µL)	9.11 ± 0.21	10.5 ± 0.55	7.95 ± 0.55	7.30 ± 0.40	7.90 ± 0.40	10.6 ± 0.90	12.2 ± 0.2	13.8 ± 1.05*
Lymph%	83.4 ± 0.10	83.7 ± 0.70	80.6 ± 0.40	85.3 ± 0.35	54.8 ± 1.50	83.8 ± 0.45***	75.6 ± 6.5***	47.3 ± 4.70***
Mid%	6.20 ± 0.20	5.05 ± 0.55	8.70 ± 0.30	5.50 ± 0.40	15.3 ± 2.20	5.25 ± 0.15	11.2 ± 0.80*	19.6 ± 0.10
Gran%	10.4 ± 0.10	11.2 ± 1.25	10.7 ± 0.70	9.15 ± 0.75	29.9 ± 0.70***	10.9 ± 0.60***	18.55 ± 1.95***	33.1 ± 4.60***
PLT								
PLT (10^3^/µL)	874 ± 45.0	842 ± 42.0	844 ± 5.50	816 ± 6.00	955 ± 53.0	1006 ± 75.0	1057 ± 93.0	802 ± 21.0
MPV (fL)	5.66 ± 0.16	5.56 ± 0.23	5.50 ± 0.30	5.98 ± 0.31	6.05 ± 0.25	5.70 ± 0.10	6.05 ± 0.15	6.25 ± 0.04
PDW (fL)	14.8 ± 0.14	15.1 ± 0.15	15.5 ± 0.5	14.7 ± 0.63	14.7 ± 0.05	14.5 ± 0.03	14.6 ± 0.05	14.5 ± 0.16
PCT (mL/L)	4.62 ± 0.08	4.08 ± 0.57	3.97 ± 0.28	4.91 ± 0.24	5.81 ± 0.54	5.72 ± 0.51	6.40 ± 0.71	5.04 ± 0.15

*N* the untreated healthy control normal mouse group, *N-CD NPs* nanocomplex of Cu_4_O_3_ with diethyldithiocarbamate (DE)-treated normal mouse group, *N-ZD NPs* nanocomplex of ZnO with DE-treated normal mouse group, *N-Fu* 5-fluorouracil-treated normal mouse group, *MT* the untreated mammary tumor-bearing mouse group, *MT-CD NPs* CD NPs-treated MT group, *MT-ZD NPs* ZD NPs-treated MT, and *MT-Fu* Fu-treated MT group, *RBC* red blood cell, *Hg* hemoglobin, *HCT* hematocrit, *MCV* mean corpuscular volume, *fL* 10^–15^ L, *MCH* mean corpuscular hemoglobin, *MCHC* mean corpuscular hemoglobin concentration, *RDW-CV and RDW-SD* red blood cell distribution width-coefficient of variation and standard deviation, *WBC* white blood cell, *Lymph* lymphocyte, *Mid* monocytes, eosinophils and basophils, *Gran* granulocyte, *PLT* platelet, *MPV* mean platelet volume, *PDW* platelet distribution width, and *PCT* plateletcrit.

All values are demonstrated as mean ± SEM. N group was compared to other groups and considered significantly different at p < 0.05*, < 0.01**, and < 0.0001***.

Regarding hematological parameters (Table 2), N-CD NPs and MT-CD NPs groups did not show any abnormal changes, relative to the healthy N group, in RBCs count, hemoglobin (Hg), and their related factors, and the total count or differential percentages of WBCs, as well as platelet (PLT) count, volume, distribution width, and plateletcrit (PCT). Both N-ZD NPs and N-Fu groups also did not demonstrate any significant hematological alterations relative to N group. On the other side, MT group revealed a significant decrease in Hg, hematocrit (HCT), and lymphocyte (Lymph) % as well as a statistical increase in red blood cell distribution width-coefficient of variation and standard deviation (RDW-CV and RDW-SD), monocyte (Mid) %, and neutrophil (Gran)% when compared to N group. In MT-Fu NPs, these abnormalities cannot be normalized by Fu which also caused a significant elevation in mean corpuscular volume (MCV) and WBCs count compared to N group. Meanwhile, MT-ZD NPs group showed a slight decrease in Hg, HCT, and Lymph% as well as a little increase in RDW-CV, RDW-SD, and neutrophil (Gran)% with a significant increase in Mid% (Table 2).

## Discussion

Worldwide, breast cancer is the most common malignancy-related death in women. The survival rate decreases dramatically, among patients with metastatic tumor, mainly attributed to the poor response to standard therapy^1^. To the best of our knowledge, this is the first study that nanoformulated DE with Cu_4_O_3_ NPs and ZnO NPs to investigate their activities on metastatic breast tumor. The generated CD and ZD nanocomplexes had nanosizes (156.5 nm and 206.5 nm, respectively), negative zeta potential, and morphology similar to their corresponding metal oxide NPs (semi-sphere and rods, respectively). In vitro anticancer assessment revealed that CD NPs was the most potent growth inhibitory activity against metastatic breast cancer cells (MDA-MB 231) with the lowest IC_50_ (31.93 μg/mL), and the highest proapoptotic and anti-metastatic potentials compared to ZD NPs and Fu. Moreover, CD NPs demonstrated the highest antitumor effect in terms of reducing mammary tumor volume and weight as well as histological, immunohistochemical, biochemical, and molecular analyses of oncogenic and apoptotic markers in mammary tumor and liver tissues. These current in vitro and in vivo demonstrations of the strongest anticancer potency of CD NPs are mostly linked to their pro-oxidant activity, as evidenced by the strongest ALDH1A inhibition in MDA-MB 231 cells and the highest ROS fold increment with GSH depletion in tumor tissues (mammary and liver) (Figs. 2a, 4c,d). Previous studies illustrated the oxidant activity of DE via its high thiol affinity, forming disulfide adducts in enzymatic and nonenzymatic antioxidant (such as ALDH1 and GSH, respectively)^28,29^. Herein, the anticancer activity-dependent pro-oxidant activity of DE was potentiated by metal oxide NPs and vice versa, as evidenced by lowering IC_50_ values of both nanocomplexes compared to DE or metal oxide NPs.

The powerful inducible oxidative stress after treatment with CD NPs rather than ZD NPs is primarily attributed to the nature of the contained metal oxide NPs (Cu_4_O_3_ and ZnO, respectively), as well as oxygen intensity that was higher in CO NPs than ZO NPs by 8.98% based on EDX analysis. Regarding CO NPs of CD nanocomplex, Cu_4_O_3_ is copper (I, II) oxide mineral with formula (Cu^+1^)_2_ (Cu^+2^)_2_ O_3_. In contrast to Cu_2_O’s direct hydroxyl radical generation effect, CuO’s production of hydroxyl radical requires GSH for reducing Cu^+2^ to Cu^+1^ which catalyzes Fenton reaction, leading to GSH depletion^30,31^. The decreased GSH is also related to the direct binding of Cu to its thiol group and the oxidative effect of the generated hydroxyl radicals, resulting in an increased formation of its oxidized form (GSSG) and, subsequently, loss of its function that affects negatively on the total cellular antioxidant capacity^31^. Cu has a higher binding affinity with greater stability constant for thiol groups than Zn^32^. Moreover, in consistence with the current findings, a previous comparison study found that CuO caused more oxidative DNA damage with extensive mitochondrial depolarization than ZnO in A549 cells^26^. The GSH lowering and ROS overproduction effects of these metal oxide NPs caused severe oxidative stress (oxidation of DNA, protein, and lipid) leading to selective cancer cell death with minimal toxicity to normal cells^30,33^. This selectivity is mainly due to their nanosize as well as abnormal tumor vascular (dilated and disorganized vessels) which enhance the permeability and retention effect of NPs selectively in tumor tissues while halting their penetration into normal vessels of healthy tissues^34^. As it was demonstrated, both nanocomplexes elevated ROS and lowered GSH only in tumor tissues (mammary and liver) without any significant changes between the treated normal tissues and healthy control untreated tissues. Furthermore, no marked variation in histology analysis and biochemical parameters was observed between N, N-CD NPs and N-ZD NPs groups. Obviously, the biodistribution of both nanocomplexes was recorded mostly (85.74% and 63.95%, respectively) in tumor tissues with only minimal percentages in normal tissues. This also indicates that CD NPs had significantly higher tumor uptake than ZD NPs (p < 0.01), which could be attributed to CD NPs’ smaller size, lesser negative charge, and semi-spherical shape. It is well understood that NPs with smaller sizes, spherical shapes, and slight negative charge achieve better circulation and tumor accumulation^34,35^.

Accordingly, CD NPs exhibited stronger selective oxidant activity, with higher tumoral uptake, which is the main contributor to inducing selective apoptosis coupled with inhibiting stemness and metastasis, higher than ZD NPs (Figs. 4, 5, 6). Overgeneration of ROS without covering by the cellular antioxidant system causes p53 expression- and activation-dependent DNA damage and mitochondrial membrane potential loss, leading to a downregulation of BCL2 and release of cytochrome C from mitochondria, and ultimately, inducing caspase-dependent apoptosis. Furthermore, GSH lowering causes apoptosis induction due to the loss of S-glutathiolated protection of GSH for caspase 3 from proteolytic cleavage activation^36^. There is a strong correlation between ROS detoxify system (e.g., ALDH1 and GSH) and CSC metastatic and self-renewal potentials. Low ROS level is critical for CSC maintenance by enhancing PI3K/AKT/mTOR activation of HIF1α-mediated hypoxia microenvironment. HIF is a crucial enhancer of GSH synthesis, glycolytic genes, Notch signaling pathway, and expression of CD44, VEGF, and ABCG2 leading to the stimulation of stemness, angiogenesis, and chemoresistance^37,38^. The latter is also induced by the overexpression of ALDH1 as well as the promotion of stemness and metastasis pathways (including, Notch1, Wnt/β-catenin, and EMT)^39^. Previous studies found that CO NPs and disulfiram-Cu complex induced ROS-mediated apoptosis by suppressing AKT signaling pathway^40,41^. HIF-1α suppression with uncontrolled ROS production leads to the elimination of CSC (metastatic seed) with blocking its stemness and metastasis potentials and restoring its sensitivity to chemotherapy^4,6,38^. In line with these previous findings^6,30^, the strongest oxidant impact of CD NPs with potent suppressing HIF-1α was linked to the lowest percentage of CD44^+^ and Ki-67^+^ immunostaining and the highest downregulation of stemness, metastasis, and VEGF genes, compared to ZD NPs and Fu (Figs. 5a–c, 6a,b). Thus, oxidant-mediated apoptosis limits tumor transformation and metastasis, as confirmed by histological analysis of the marked elimination of mammary tumor cells with complete inhibition of their metastasis to liver or lung, and immunostaining Ki-67 decline with the lowest MMP9 expression and hepatic tumor marker (AFP) level in MT-CD NPs group, compared to MT-ZD NPs and MT-Fu. Moreover, CD NPs preferentially eradicated metastatic tumor without any alterations in normal values of liver or kidney functional indicators and hematological parameters, as demonstrated in Tables 1 and 2.

In conclusion, these new nanoformulations of DE with green chemically synthesized CO NPs (Cu_4_O_3_) and ZO NPs exhibited a selective oxidant potential-dependent anticancer potency, compared to Fu, using metastatic breast cancer cells (MDA-MB 231) and metastatic mammary tumor animal model. Importantly, CD NPs demonstrated higher tumoral uptake and stronger oxidant activity than ZD NPs. This massive oxidative stress in MT-CD NPs was associated with the strongest HIF1α suppression, caspase 3-dependent apoptosis induction, and CD44^+^CSCs (seeds of metastasis) elimination with blocking their stemness, chemoresistance, and metastasis gene expression compared MT-ZD NPs and MT-Fu. Hence, histological analysis and immunostaining with Ki-67 and caspase 3 as well as lowering MMP-9 expression and liver tumor marker (AFP) level confirmed that CD NPs had the best therapeutic potential for the eradication of metastatic mammary tumor (mixed ductal and lobular mammary carcinoma), compared to ZD NPs and Fu. Besides that, CD NPs did not show any marked variation in the functional parameters of liver and kidney as well as hematological indexes, compared to healthy control group. Collectively, this novel nanocomplex of DE and Cu_4_O_3_ NPs was more effective than ZD nanocomplex in annihilating metastatic breast cancer.

## Materials and methods

### Materials

Chitosan was obtained from Acros Organics (New Jersey, USA). Dulbecco’s modified Eagle’s medium (DMEM) and fetal bovine serum (FBS) were obtained from GIBCO (New York, USA). Standard chemotherapy (fluorouracil) was from Applichem for pharmaceuticals Co. (Darmstadt, Germany). 3-(4,5-dimethylthiazol-2-yl)-2,5-diphenyl tetrazolium bromide (MTT), retinal, 2,7 dichlorofluorescence diacetate (DCFDA), nuclear stains, horseradish peroxidase-conjugated secondary antibody, 3,3′ diaminobenzidine, glutathione (GSH) were purchased from Sigma-Aldrich (Burlington, Massachusetts, USA). Trypan Blue, primary antibodies of ki-67, CD 44, and caspase 3, RNA extraction kit, and primers were obtained from Thermo Fisher Scientific (Waltham, Massachusetts, USA). One-step qPCR SYBR green master mix kit and α-Fetoprotein electrochemiluminescence detection kit were from GeneDireX (Taoyuan, Taiwan) and Roche Diagnostics (Basel, Switzerland), respectively.

### Methods

#### Preparation and characterization of NPs

Copper oxide nanoparticles were prepared by mixing copper chloride (source of copper salt) with chitosan (capping agent), and vitamin C (reducing agent) as recently described^27^. ZO NPs were prepared using the precipitation method^42^ with some modifications, including zinc chloride (0.2 mL, 160 mM) was mixed with NaOH (10 mL, 1 M) and vitamin C (10 mL, 20%), pH was adjusted to 7, and heated for 1 h at 80 °C. Then ZO NPs were obtained after centrifugation. Zetasizer Nano ZS (Malvern, UK) was used to assess the size and zeta potential. Compositional feature, oxidation state (structural property), and morphology were determined using energy dispersive X-ray analysis (EDX, JEOL JEM-1230, Japan), X-ray diffractometer (XRD, Bruker MeaSrv (D2-208219) fitted with Cu Kα radiation tube (λ = 1.5406 Å)^27^, and scanning electron microscopes (SEM, JEOL JSM (5300), Japan)^27^, respectively. The latter instrument's data was analyzed utilizing Joint Committee on Powder Diffraction Standards (JCPDS).

These characterized NPs (0.02 mg/mL) were mixed with DE (0.2 mg/mL) to form nanocomplexes of CD NPs and ZD NPs, respectively. The size, zeta potential, and morphology of these nanocomplexes were then determined as mentioned above. In parallel, traditional complexes of copper chloride-DE (CD) and zinc chloride-DE (ZD) were prepared by the same ratio.

#### In vitro evaluation of anti-breast cancer activity

##### Determination of cancer growth inhibition by MTT assay

Human breast cancer cell line (MDA-MB 231) which was obtained from American Type Culture Collection (ATCC), seeded (5 × 10^3^ cells, passage no. 41) in 96-well cell culture plates using DMEM medium with 10% FBS as the culture medium, and incubated in 5% CO_2_ incubator at 37 °C. After 24 h, the attached cancer cells were treated with serial concentrations of CD NPs, ZD NPs, CD, ZD, and standard chemotherapy (fluorouracil) as well as DE, copper chloride and zinc chloride. Following 48 h in 5% CO_2_ incubator, MTT (5 mg/mL) was incubated with the untreated and treated cells for 4 h then DMSO was added and the absorbance was read at 590 nm^43^ using spectrophotometer plate reader (BMG LabTech, Germany). The IC_50_ value, at which 50% cancer growth inhibition, was estimated by GraphPad Prism 9. Additionally, a change in the morphology of the treated breast cancer cell was observed using phase contrast inverted microscope with a digital camera (Olympus, Japan).

##### Flow cytometry analysis of apoptosis with morphological detection using fluorescence microscopy

After 48 h treatment of MDA-MB 231 with CD NPs, ZD NPs, CD, ZD, and fluorouracil (Fu) at the lowest IC_50_ (30 μg/mL), these cells were trypsinized, washed, and incubated for 15 min with fluorescein isothiocyanate (FITC)-annexin V/propidium iodide (PI). The percentage of annexin V-stained apoptotic cells was assessed by FITC signal sensor (FL1) versus the phycoerythrin emission signal sensor (FL2)^44^. Moreover, fluorescence microscope was used after staining adherent MDA-MB 231 cells with acridine orange and ethidium bromide (AO/EB) to confirm the induction of apoptosis in the treated cells.

##### Investigation of anti-migration efficacy by wound-healing assay

After MDA-MB 231 had reached 90% confluence, the monolayer was scratched and treated with safe doses of the prepared nanocomplexes or traditional complexes (at ~ 0.1 μg/mL). At 0 h and 24 h, the wound area was photographed and assessed by image j software to calculate the percentage inhibition of migration in the treated versus untreated wells.

##### Determination of ALDH1 activity inhibition

The inhibitory potency (%) of the tested complexes on ALDH1 activity was determined by first incubating (10 min) the untreated and treated cell lysates with 500 µM NAD+ (ALDH cofactor) at 37 °C. Then to initiate ALDH reaction, retinal (substrate) was added. The ALDH activity was assessed as the rate of NADH generation/min/total protein level at 340 nm^45^ using microplate spectrophotometer (BMG LabTech, Germany). Cellular protein content was quantified using Bradford assay^46^.

#### In vivo investigation of anti-metastatic breast tumor potential

##### Experimental design

The current animal study was conducted upon the agreement of Institutional Animal Care and Use Committee (Alex-IACUC)-member of International Council for Laboratory Animal Science (ICLAS) with approval number AU-0122172832. In accordance with ARRIVE guidelines, all applicable national and international rules for the use of experimental animals were followed.

Ehrlich ascites tumor cells were obtained from frozen aliquots, in liquid nitrogen, then subcultured by intraperitoneal inoculations in BALB/c female mice. The cell viability and count were checked using Trypan Blue exclusion method. The inoculum concentration was adjusted to 2 × 10^6^ cells/0.2 mL phosphate-buffered saline (PBS). The metastatic mammary tumor which was induced by a single injection of Ehrlich cells (2 × 10^6^) in thoracic mammary fat pad of 40 BALB/c female mice (16–20 g), was confirmed by histological examination of liver tissues of 4 mice after 2 weeks (Fig. 3a). Thirty-six mice were blindly divided into 4 groups (9 mice/group), including untreated mammary tumor-bearing group (MT), CD NPs (2 mg/kg)-treated MT group (MT-CD NPs), ZD NPs (2 mg/kg)-treated MT group (MT-ZD NPs), and fluorouracil (10 mg/kg)-treated MT group (MT-Fu). Three treated MT groups were injected intravenously (i.v.) three times a week for two weeks. Mammary tumor volume ([tumor width^2^ × tumor length]/2) was measured, 3 times/week, using a caliper as well as body weight was recorded 3 times/week.

In parallel, 36 healthy normal mice (16–19 g) were also blindly divided into 4 groups (9 mice/group), including normal healthy (N) untreated group, CD NPs (2 mg/kg)-treated group (N-CD NPs), ZD NPs (2 mg/kg)-treated group (N-ZD NPs), and Fu (10 mg/kg)-treated group (N-Fu).

At the end of the experiment (two mice of MT group died), animals were anaesthetized until their respiration ceased by placing them in a tightly closed chamber containing 5% isoflurane. Then the excised tumors and other tissues (liver, lung, brain, and kidney) were collected. Mammary tumors were weighted for estimating the percentage reduction in tumor growth of the treated groups relative to MT group as well as liver weight was measured. Also, blood was collected in heparin and EDTA tubes for assessing the biochemical parameters and complete blood count (CBC), respectively. Part of the tissues was fixed in 10% formalin for histological and immunohistochemical analyses, while the remainder was stored at – 80 °C for biochemical and molecular analyses.

##### Histological examinations

The fixed tissues were prepared according to the standard histological protocol. Briefly, these tissues were dehydrated, embedded in paraffin and cut into micrometer sections. Regarding histological analysis, these sections were transferred into traditional slides for staining with hematoxylin and eosin (H&E)^47^.

##### Atomic absorption spectrometric quantification of biodistribution of CD NPs and ZD NPs

In a separate animal study, ten MT mice were divided, after 4 weeks of orthotopic injection with Ehrlich cells, into two groups (5 mice/group) which were i.v. injected with 0.1 mg CD NPs and 0.1 mg ZD NPs. After 1 and 4 h, blood was collected from the orbital sinus of isoflurane-anesthetized mice. The next day, animals were sacrificed then blood, thoracic mammary tumor, normal inguinal mammary glands, liver, lung, brain, heart, and kidney were collected. Then copper and zinc which correspond to CD NPs and ZD NPs, respectively, were then quantified, in the digested tissues, using graphite atomic absorption (Analytik Jena AG, Germany). After normalization with the detected concentrations of copper and zinc in tissues of 5 untreated MT mice (as blank), the tissue uptake % was assessed relative to the original injected amounts.

##### Quantification of tissue ROS and reduced glutathione (GSH) levels

The level of ROS was assessed by incubating DCFDA (10 μM) with 100 μL homogenate (0.1 mg in 1 mL of 40 mM Tris–HCl buffer pH 7.4) of mammary and liver tissues of the untreated (MT and N) and all treated groups. After 40 min, the fluorescence intensity of DCF was measured with fluorometer plate reader (BMG LabTech, Germany) at excitation 485 nm and emission 525 nm^48^.

The GSH level was determined after incubation of tissue homogenates with Ellman's reagent (5,5′-dithiobis2-nitrobenzoic acid). After 10 min, the absorbance of the generated yellow-colored product was measured with spectrophotometric plate reader (BMG LabTech, Germany) at 412 nm^49^. The total protein level was determined by Bradford assay^46^.

##### Immunohistochemical analyses

The fixed tissues were prepared based on the typical immunohistochemistry protocol. Mammary tumor and liver tissue sections were transferred into positively charged slides that had been dried, dewaxed, boiled in citrate buffer (10 mM, pH 6). Following cooling, these slides were incubated separately with primary antibodies of ki-67, CD 44, and caspase 3. After washing and incubating with horseradish peroxidase-conjugated secondary antibody then washing and adding substrate (3,3′ diaminobenzidine), brown-colored product was formed^50^, recorded by CellSens imaging analysis software of phase-contrast microscope, and analyzed using image j software.

##### Assessment of anti-metastatic activity via quantification of liver tumor marker

The common liver tumor marker (α-fetoprotein, AFP) was measured in blood samples of the untreated and treated MT groups using the electrochemiluminescence detection kit method.

##### Relative change in the expression of tumor suppressor gene and oncogenes

The untreated and treated MT groups’ mammary tumor and liver tissues were homogenized in lysis buffer (supplied in kit) with β-mercaptoethanol then total RNA was extracted according to the standard protocol described in the kit. Following RNA purification and quantification, one-step qPCR SYBR green master mix kit was used with the specific primers (Supplementary Table 1) to assess the relative change in the gene expression of p53, BCl2, HIF-α, β-Catenin, Notch1, ATP Binding Cassette Subfamily G Member 2 (ABCG2), VEGF, and MMP9 using equation of 2^−ΔΔCt^.

##### Liver and kidney function parameters and CBC

Parameters’ liver function (ALT, AST and albumin) and kidney function (urea and creatinine) were detected in plasma using colorimetric kits as well as complete blood count (CBC) was determined using a hematology analyzer (Mindray, China).

### Statistical analysis

Data are demonstrated as mean ± standard error of mean (SEM) and were analyzed by GraphPad Prism 9.3.1. using one-way analysis of variance (ANOVA)-multiple comparison (Dunnett test) and significance was considered at p < 0.05*, < 0.01**, and < 0.001***.

## Supplementary Information

Supplementary Figures.

Supplementary Table 1.

## Data Availability

The original data and materials used in the study are available upon reasonable request from the corresponding authors.
