# Supplementary material for: A comparative study of smart nanoformulations of diethyldithiocarbamate with Cu4O3 nanoparticles or zinc oxide nanoparticles for efficient eradication of metastatic breast cancer
Source: Sci Rep. 2023 Mar 2;13:3529. doi: 10.1038/s41598-023-30553-8 (PMC9981580; doi:10.1038/s41598-023-30553-8)
Supplement: Supplementary file 1 — Supplementary Figures. [file 41598_2023_30553_MOESM1_ESM.docx]

**A comparative study of smart nanoformulations of diethyldithiocarbamate with Cu_4_O_3_ nanoparticles or zinc oxide nanoparticles for efficient eradication of metastatic breast cancer**

**Marwa M Abu-Serie^a*^, Eisayeda Zeinab A Abdelfattah^b^**

^a^Medical Biotechnology Department, Genetic Engineering and Biotechnology Research Institute, (GEBRI), City of Scientific Research and Technological Applications (SRTA-City), New Borg El‑Arab City, Alexandria 21934, Egypt. **Phone:** +2034593422 **Fax:** +2034593407

*Correspondence: [marwaelhedaia@gmail.com](mailto:marwaelhedaia@gmail.com)

^b^Animal House Unit, Medical Technology Center, Medical Research Institute, Alexandria University, Alexandria, Egypt. [Zeinab.abdelfattah@yahoo.com](mailto:Zeinab.abdelfattah@yahoo.com)

**
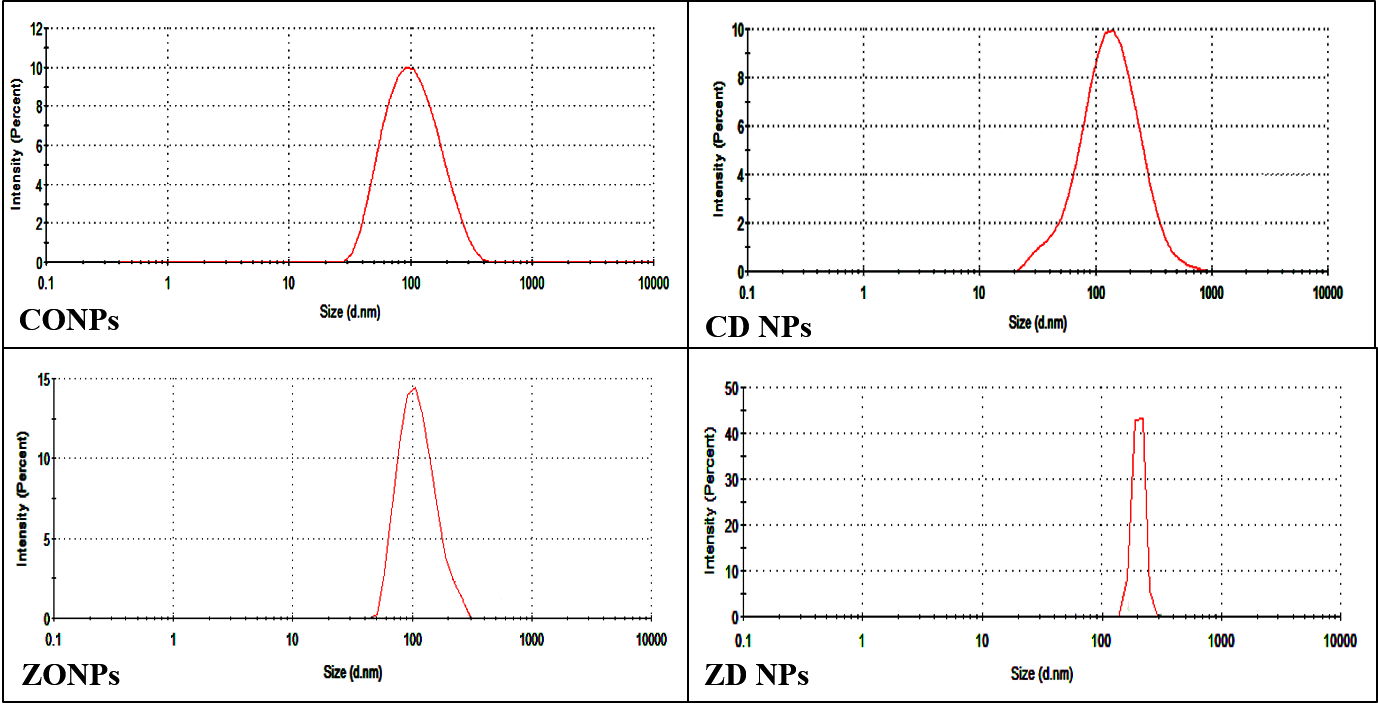
**

**Supplementary Figure 1. Nanosizes of CO NPs and ZO NPs and their nanocomplexes with diethyldithiocarbamate.**

**
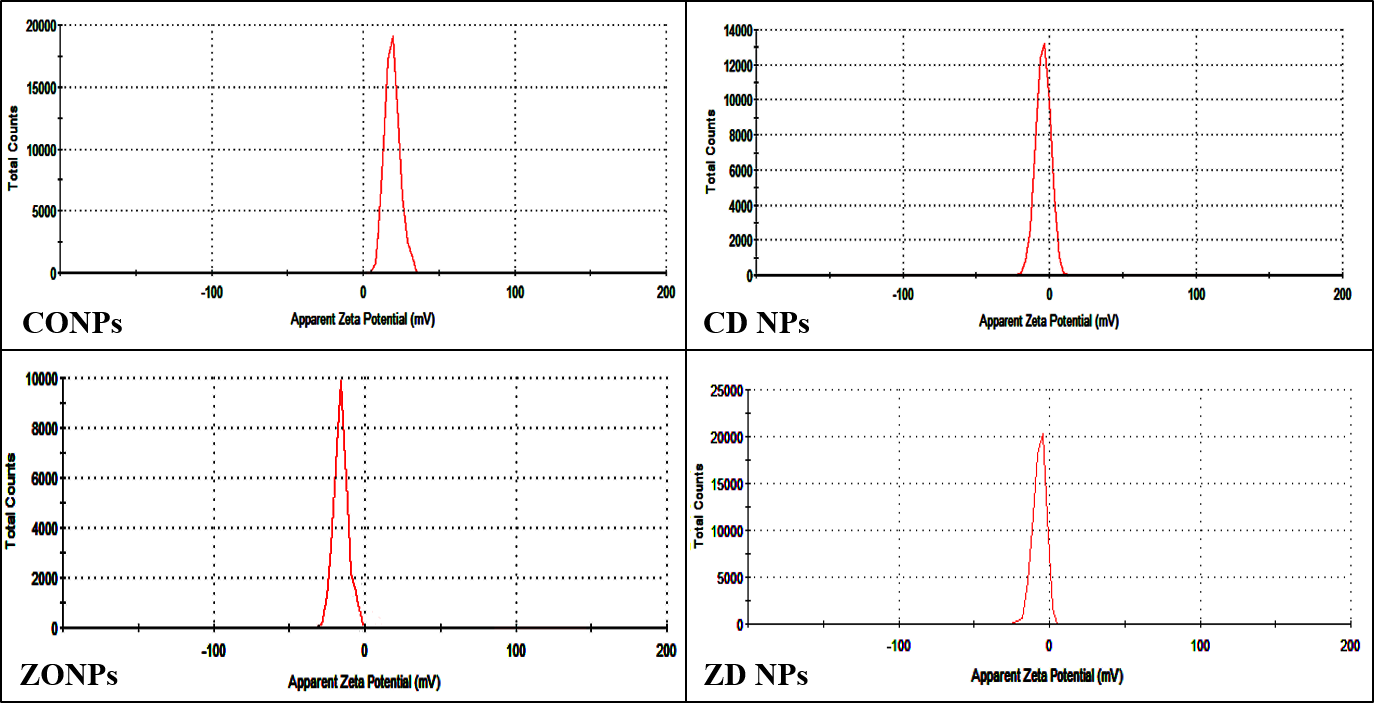
**

**Supplementary Figure 2. Zeta potentials of CO NPs and ZO NPs and their nanocomplexes.**
