# Supplementary material for: A comparative study of smart nanoformulations of diethyldithiocarbamate with Cu4O3 nanoparticles or zinc oxide nanoparticles for efficient eradication of metastatic breast cancer
Source: Sci Rep. 2023 Mar 2;13:3529. doi: 10.1038/s41598-023-30553-8 (PMC9981580; doi:10.1038/s41598-023-30553-8)
Supplement: Supplementary file 2 — Supplementary Table 1. [file 41598_2023_30553_MOESM2_ESM.docx]

**A comparative study of smart nanoformulations of diethyldithiocarbamate with Cu_4_O_3_ nanoparticles or zinc oxide nanoparticles for efficient eradication of metastatic breast cancer**

**Marwa M Abu-Serie^a*^, Eisayeda Zeinab A Abdelfattah^b^**

^a^Medical Biotechnology Department, Genetic Engineering and Biotechnology Research Institute, (GEBRI), City of Scientific Research and Technological Applications (SRTA-City), New Borg El‑Arab City, Alexandria 21934, Egypt. **Phone:** +2034593422 **Fax:** +2034593407

*Correspondence: [marwaelhedaia@gmail.com](mailto:marwaelhedaia@gmail.com)

^b^Animal House Unit, Medical Technology Center, Medical Research Institute, Alexandria University, Alexandria, Egypt. [Zeinab.abdelfattah@yahoo.com](mailto:Zeinab.abdelfattah@yahoo.com)

**Supplementary Table 1. Forward (F) and reverse (R) primers of the studied genes**

| **Gene** | **Primers** |
| --- | --- |
| p53 | F: 5′-CACAGCACATGACGGAGGTC-3′  R: 5′-TCCTTCCACCCGGATAAGATG-3′ |
| BCl2 | F: 5′-GAGAGCGTCAACAGGGAGATG-3′  R: 5′-CCAGCCTCCGTTATCCTGGA-3′ |
| HIF-α | F: 5′-GATGACGGCGACATGGTTTAC-3′  R: 5′-CTCACTGGGCCATTTCTGTGT-3′ |
| β-Catenin | F: 5′-ATGGAGCCGGACAGAAAAGC-3′  R: 5′-TGGGAGGTGTCAACATCTTCTT-3′ |
| Notch1 | F: 5′-CCCTTGCTCTGCCTAACGC-3′  R: 5′-GGAGTCCTGGCATCGTTGG-3′ |
| ABCG2 | F: 5′-GAACTCCAGAGCCGTTAGGAC-3′  R: 5′-CAGAATAGCATTAAGGCCAGGTT-3′ |
| VEGF | F: 5′-GTGAGGTGTGTATAGATGTGGGG-3′  R: 5′-ACGTCTTGCTGAGGTAACCTG-3′ |
| MMP9 | F: 5′-GCGTCGTGATCCCCACTTAC-3′  R: 5′-CAGGCCGAATAGGAGCGTC-3′ |

(HIF)1α; hypoxia-inducible factor, ABCG2; ATP Binding Cassette Subfamily G Member 2, VEGF; vascular endothelial growth factor, MMP9; matrix metalloprotease 9.
